# Both Ligand- and Cell-Specific Parameters Control Ligand Agonism in a Kinetic Model of G Protein–Coupled Receptor Signaling

**DOI:** 10.1371/journal.pcbi.0030006

**Published:** 2007-01-12

**Authors:** Tamara L Kinzer-Ursem, Jennifer J Linderman

**Affiliations:** 1 Department of Chemical Engineering, University of Michigan, Ann Arbor, Michigan, United States of America; 2 Department of Biomedical Engineering, University of Michigan, Ann Arbor, Michigan, United States of America; Purdue University, United States of America

## Abstract

G protein–coupled receptors (GPCRs) exist in multiple dynamic states (e.g., ligand-bound, inactive, G protein–coupled) that influence G protein activation and ultimately response generation. In quantitative models of GPCR signaling that incorporate these varied states, parameter values are often uncharacterized or varied over large ranges, making identification of important parameters and signaling outcomes difficult to intuit. Here we identify the ligand- and cell-specific parameters that are important determinants of cell-response behavior in a dynamic model of GPCR signaling using parameter variation and sensitivity analysis. The character of response (i.e., positive/neutral/inverse agonism) is, not surprisingly, significantly influenced by a ligand's ability to bias the receptor into an active conformation. We also find that several cell-specific parameters, including the ratio of active to inactive receptor species, the rate constant for G protein activation, and expression levels of receptors and G proteins also dramatically influence agonism. Expressing either receptor or G protein in numbers several fold above or below endogenous levels may result in system behavior inconsistent with that measured in endogenous systems. Finally, small variations in cell-specific parameters identified by sensitivity analysis as significant determinants of response behavior are found to change ligand-induced responses from positive to negative, a phenomenon termed protean agonism. Our findings offer an explanation for protean agonism reported in β_2_--adrenergic and α_2A_-adrenergic receptor systems.

## Author Summary

G protein–coupled receptors (GPCRs) are transmembrane proteins involved in physiological functions ranging from vasodilation and immune response to memory. The binding of both endogenous ligands (e.g., hormones, neurotransmitters) and exogenous ligands (e.g., pharmaceuticals) to these receptors initiates intracellular events that ultimately lead to cell responses. We describe a dynamic model for G protein activation, an immediate outcome of GPCR signaling, and use it together with efficient parameter variation and sensitivity analysis techniques to identify the key cell- and ligand-specific parameters that influence G protein activation. Our results show that although ligand-specific parameters do strongly influence cell response (either causing increases or decreases in G protein activation), cellular parameters may also dictate the magnitude and direction of G protein activation. We apply our findings to describe how protean agonism, a phenomenon in which the same ligand may induce both positive and negative responses, may result from changes in cell-specific parameters. These findings may be used to understand the molecular basis of different responses of cell types and tissues to pharmacological treatment. In addition, these methods may be applied generally to models of cellular signaling and will help guide experimental resources toward further characterization of the key parameters in these networks.

## Introduction

G protein–coupled receptors (GPCRs) are the largest class of cell membrane receptors with almost 2,000 members identified [1]. It is estimated that more than 50% of pharmaceuticals target GPCRs [2]. While the majority of pharmacologic research has focused on ligand-specific properties that influence cell behavior, relatively few studies focus on cell-specific parameters that may also determine cell responses [3–5]. Studying the effect of changes in both ligand- and cell-specific parameters on cellular behavior is complicated by the large number of interactions and feedback mechanisms inherent in cellular signaling. Thus it becomes necessary to use quantitative models to aid in the analysis of these systems.

**Figure 1:**
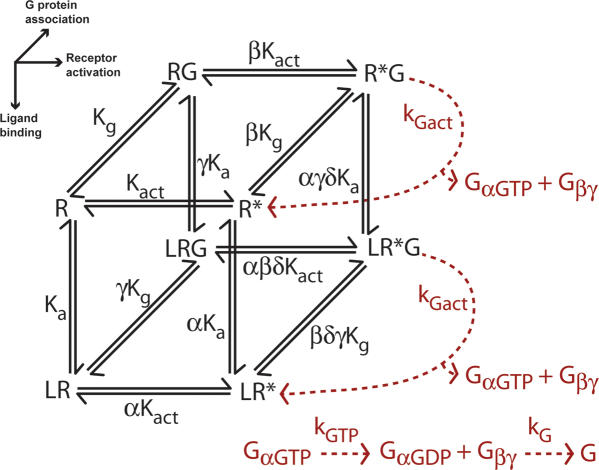
The Cubic Ternary Complex Model and the Cubic Ternary Complex Activation Model The cTCM (black) is a thermodynamically complete equilibrium representation of ligand (L), receptor (R), and G protein (G) interactions [11]. Association and dissociation of L and R is represented here from top to bottom, R and G interactions from front to back, and the interconversion of inactive and active R states from left to right of the cube. Based on the cTCM, the cTCAM (black and red) incorporates the dynamics of activation and recycling of G protein (dashed lines) into a kinetic model of LRG interactions [22]. A brief summary of model parameters is found in Table 1. Description of model parameters, assumptions, and equations are given in Text S1.

Typical models of GPCR signaling are termed ternary complex models or TCMs (reviewed in [6–8]). These models feature ligand (L) binding to receptor (R) to form a ligand–receptor complex (LR), and LR interaction with G protein (G) to form the ternary ligand–receptor–G protein (LRG) complex. Subsequent equilibrium models of GPCR signaling have remained true to this paradigm while incorporating additional receptor (e.g., active receptor R* or inactive receptor R) or G protein states or other effectors to account for experimental findings [9–15]. A key feature of these models is that active receptors can associate with G protein in the absence or presence of ligand to form R*G and LR*G, respectively, and both these complexes can signal. Kenakin and colleagues have proposed a thermodynamically complete representation of ligand, receptor, and G protein interactions termed the cubic ternary complex model (cTCM) and shown in Figure 1 [11]. In this model, inactive receptor can both bind ligand (to form LR) and associate with G protein (to form RG or LRG).

**Table 1:**
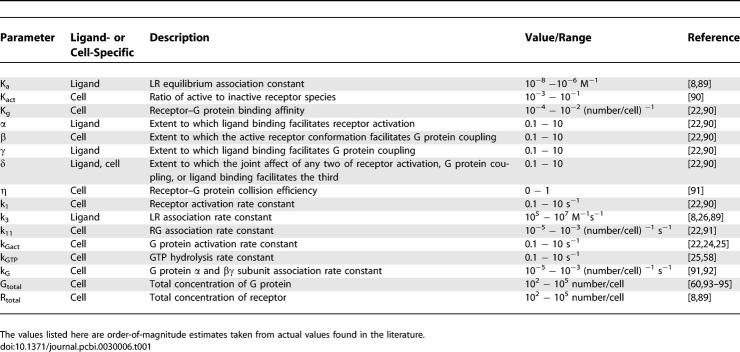
The 16 Independent Parameters of the cTCAM

TCMs are typically equilibrium models and while they have been widely used, it is well known that kinetic models are better able to replicate the intrinsic dynamics of signal transduction, as has been discussed previously (see [14,16–20]). Furthermore, predictions of kinetic and equilibrium models with similar parameter values can be markedly different [21,22]. Indeed, a number of groups have discussed the importance of kinetics in analyzing GPCR systems [8,20,23–27]. Our group has thus developed a kinetic version of the cTCM termed the cubic ternary complex activation model (cTCAM, Figure 1) [22]. The cTCAM incorporates a G protein activation feedback loop whereby G proteins (G) couple to and are activated by active receptors (R* and LR*), allowing for GTP binding and uncoupling of the G protein heterotrimer into α and βγ subunits. The GTP on active G proteins (Gα_GTP_) is hydrolyzed by the intrinsic GTPase activity of the alpha subunit (with or without participation of regulator of G protein signaling (RGS) proteins) to form inactive Gα_GDP_ subunits. The feedback loop is completed when the Gα_GDP_ and Gβγ subunits couple to reform inactive G protein (G). We found that the predictions of the kinetic model (cTCAM) can be strikingly different than those of the equilibrium model (cTCM) in terms of the character of the response (positive/neutral/inverse agonism), suggesting the importance of using the more realistic dynamic model [22].

In this work we use the cTCAM as a dynamic model of the initial events in GPCR signaling to identify both the ligand- and cell-specific parameters that may be key determinants of the character of cellular response. One drawback of the kinetic cTCAM, and indeed for most if not all signal transduction models, is that many parameter values have not been measured and others may vary over several orders of magnitude [10,12,22,25,28]. Additionally, the large number of parameters and incorporation of the G protein activation feedback loop make intuition of model behavior difficult. Thus it becomes necessary to introduce techniques for efficient sampling of the input parameter space and quantification of model output. In the risk analysis and environmental engineering fields, uncertainty and sensitivity analysis have been routinely used to sample input parameter space and identify key parameters [29,30]. These techniques have recently been introduced into the biological sciences. In particular, Latin hypercube sampling (LHS) with partial rank correlation coefficients (PRCC) has been used to perform uncertainty and sensitivity analysis in epidemiological studies of HIV and tuberculosis [31,32], to understand the dynamics of tuberculosis infection and immunity in host–pathogen models [33,34], and to analyze parameter sensitivity in a T cell receptor activated Erk–MAPK signaling pathway [35]. Additionally, several studies have used genetic algorithm-based search methods to perform parameter fitting and sensitivity analysis for models of T cell receptor and GPCR activation [12,35,36]. Rundell and colleagues found that these global analysis methods (LHS/PRCC and genetic algorithm approaches) and others (Sobol's method and Fournier amplitude sensitivity test (FAST)) give very similar results [35].

LHS has been shown to be a computationally efficient method for sampling parameter ranges. It is more than one order of magnitude more efficient than random sampling methods [29,30,37]. Additionally, statistical techniques can be used to identify parameters that are most important in determining output variables. Correlation coefficients can be readily calculated to identify parameters whose variation is strongly correlated with variations in an output parameter of interest. For nonlinear monotonic systems such as the cTCAM, PRCC is known to be the most appropriate [29,38,39]. PRCC values can be calculated at each time point of the simulation, and the relative importance of the parameters can be tracked over time. Here we use LHS and PRCC to identify parameters that are important determinants of G protein activation in a general model of GPCR signaling (cTCAM, Figure 1). In particular, we are interested in how small variations in parameter values might give rise to large differences in the character (positive/neutral/inverse agonism) of ligand-induced responses.

Different ligands, while binding to the same receptors, are often able to transmit different levels of signal per bound receptor and thus have different levels of response. Recent studies have shown that the same ligand may not only induce varying levels of response but also both positive and negative responses in some GPCR systems. Here we refer to the ability of a ligand to act both as a positive agonist and an inverse agonist as protean agonism (a term previously introduced by Kenakin [40]). Protean agonists (ligands that are able to induce protean agonism) have been identified in several GPCR systems, including β_2_-adrenergic, α_2A_-adrenergic, opioid, and histamine H3 receptors [41–44]. After identification of ligand- and cell-specific parameters that play critical roles in determining the character of a response, we then focus on two particularly interesting studies of β_2_-adrenergic and α_2A_-adrenergic receptors in which both positive and inverse agonism are produced by introduction of the same ligand to the system. Chidiac and colleagues reported that the β_2_-adrenergic receptor partial agonist dichloroisoproterenol (DCI) acted as both a positive and an inverse agonist in Sf9 cells overexpressing β_2_-adrenergic receptor despite the fact that the treatment of the cells was similar in both cases [41]. Jansson and colleagues reported that the α_2A_-adrenergic ligand levomedetomidine (levomed) had opposite effects on cAMP production in different cell lines, activating the receptor in several systems (S115 and PC10 cells) while inhibiting constitutive activity of the endogenous receptor in HEL 92.1.7 cells [42,45,46]. We find that changes in cell-specific parameters identified as key determinants of response behavior by sensitivity analysis are consistent with these seemingly contradictory behaviors in the β_2_- and α_2A_-adrenergic receptor systems.

## Results

### Cell-Specific Parameters Are Highly Correlated with Variation in Response Characteristics

**Figure 2:**
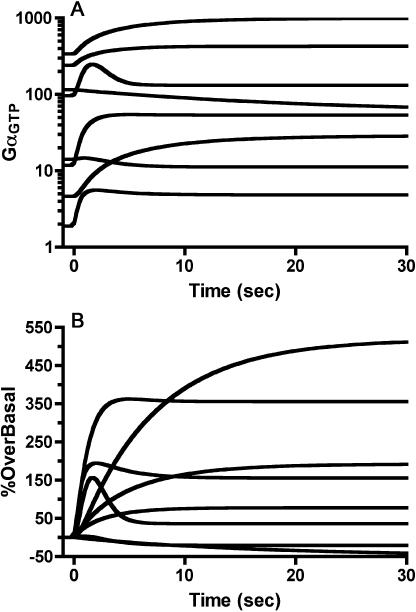
Time Course of Representative Model Outputs Parameter values are sampled using LHS, and the differential equations describing the cTCAM are solved according to the equations in Text S1. G protein activation as quantified by Gα_GTP_ is tracked over time (see Equation S.27 in Text S1). (A) Values of Gα_GTP_ (number/cell) for eight parameter sets from LHS sampling of the ranges in Table 1 are plotted over the course of the simulation. (B) Percent change in the value of Gα_GTP_ relative to basal values (%OverBasal) was calculated and tracked over time according to Equation 1. [L] = 0.1 μM.

LHS was used to efficiently sample parameter values from the ranges listed in Table 1. One LHS simulation typically sampled each of the 16 parameters 1,000 times, producing 1,000 solutions to the model equations (Text S1). The formation of Gα_GTP_ is computed (see Equation S.27 in Text S1). Eight example solutions of the time course of Gα_GTP_ formation are shown in Figure 2A. As expected, variations in parameter values caused large variations in response behavior. For example, for the cases shown in Figure 2A, at steady state prior to ligand addition (time zero), Gα_GTP_ values varied between approximately two and 240 per cell. Once ligand is added to the system, it binds to receptors which in turn bind to G protein, changing the distribution of receptors and G proteins among their various states and causing increases or decreases in G protein activation (as seen by changes in Gα_GTP_ formation, Figure 2A). In some instances, G protein activation increases rapidly, and then falls due to GTP hydrolysis. Ligand-induced responses occur on the order of 5 s to 30 s at this ligand concentration, which is approximately the timescale over which G protein activation is known to occur [47–49]. Calculating the percent change of Gα_GTP_ upon ligand addition (designated %OverBasal) allowed for easier observation of decreases in G protein activity upon ligand binding; in Figure 2B these are seen as negative %OverBasal values. In this way, responses were directly related to the pharmacological classifications of ligand efficacy—positive (increase in %OverBasal), neutral (no change in %OverBasal), and inverse agonism (decrease in %OverBasal)—and thus can be compared with experimental data that normalize to control conditions.

To determine the correlation between parameter values and levels of G protein activation, PRCC values were calculated at 0.25-s intervals for varying ligand concentrations (0.1 nM to 0.1 mM). Correlations (PRCC values) for each parameter listed in Table 1 were calculated with respect to the two different measurements (outputs) of G protein activation discussed above, the number of Gα_GTP_ and %OverBasal. Table 2 shows the rank order of PRCC values for the two responses at two time points, 5 s and 2.5 min, and two ligand concentrations, 1 nM and 10 μM, representing pre- and post-steady state time points and sub- and supra-saturating ligand conditions, respectively.

**Table 2:**
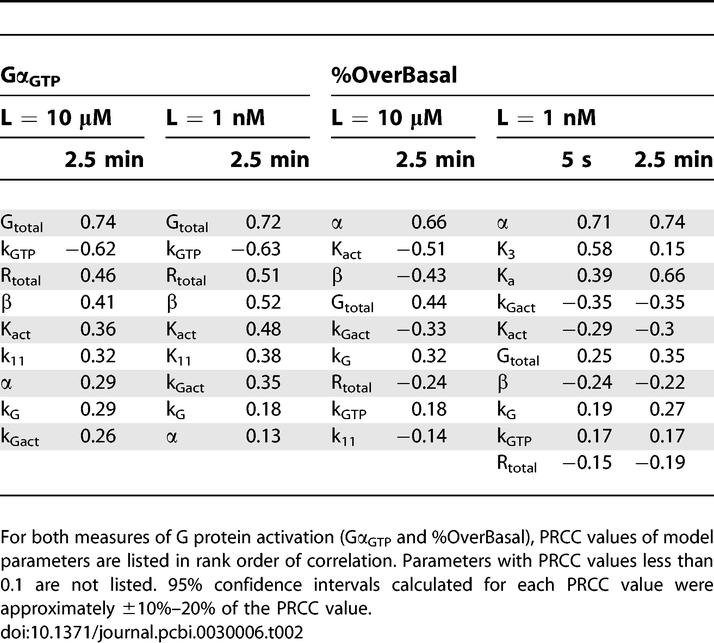
Parameters Significantly Correlated with G Protein Activation

Previous analysis of the equilibrium cTCM in our group found that K_act_, G_total_, α, δ, γ, and K_g_ all played a role in determining the character (positive/neutral/inverse agonism) of the response [20]. By using the cTCAM to include the necessary activation events, however, a somewhat different set of parameters was found to be highly correlated with response generation as summarized in Table 2. Not surprisingly, the ligand-specific parameter most correlated with both measures of response generation (instantaneous number of Gα_GTP_ and %OverBasal) was the effectiveness with which the ligand induces an active receptor conformation (α). Indeed, this was the only ligand-specific parameter found to be highly correlated with the Gα_GTP_ response. The character of responses was influenced by several cell-specific parameters including receptor and G protein expression (R_total_ and G_total_), parameters involved in the G protein activation loop (k_Gact_, k_GTP_, and k_G_), the equilibrium ratio of active to inactive receptor (K_act_), and the rate and efficiency of receptor–G protein coupling (β and k_11_). Although generally the parameters that were highly correlated did not differ between the two response measures (Gα_GTP_ and %OverBasal), the rank order of the PRCC values did vary. Similar results were found when the microscopic reversibility assumption of the model (discussed in the Methods section) was relaxed and all forward and reverse rate constants (shown in Figure S1) were varied (unpublished data).

The rank order of the parameters that were highly correlated with Gα_GTP_ did not vary significantly with time, and thus PRCC values for the parameters at 2.5 min but not 5 s are shown in Table 2. Additionally, the PRCC values did not vary significantly at varying ligand concentrations, with the exception that k_G_ and α were found to be lower in rank order at the lower ligand concentrations.

**Figure 3:**
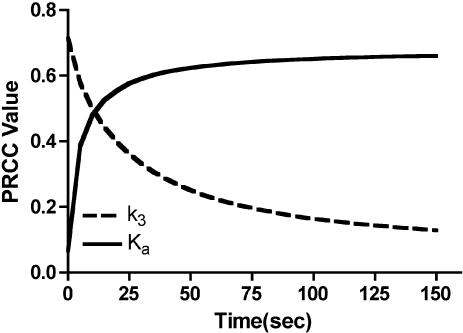
Example of the Time Course of PRCC Values Time course of PRCC values for LR association rate constant (k_3_) and LR equilibrium association constant (K_a_) correlated to %OverBasal (as given by Equation 1) when [L] = 1 nM.

For the %OverBasal response, significant differences were seen between low and high ligand concentrations. At low ligand concentration (1 nM in Table 2), the parameters for reversible ligand binding (k_3_ and K_a_) were highly correlated with %OverBasal. Additionally, the PRCC values of both k_3_ and K_a_ were found to vary with time, and thus the PRCC values for all parameters at both 5 s and 2.5 min at this ligand concentration are listed in Table 2. As shown in Figure 3, the ligand association rate constant k_3_ was found to be highly correlated at 5 s, but was not significantly correlated after 2.5 min of ligand binding. In contrast, the PRCC value for K_a_ rapidly increased over the first 30 s of the simulation and was significantly correlated with %OverBasal at longer times (greater than 1 min). Thus at sub-saturating ligand concentrations in the first few seconds of ligand binding when response characteristics are likely to be determined, changes in response are highly sensitive to the ligand association rate constant. At high ligand concentration (10 μM in Table 2), k_3_ and K_a_ were not highly correlated with %OverBasal, nor was there a significant difference between PRCC values at the two times, and thus only PRCC values at 2.5 min are listed in Table 2.

### Receptor and G Protein Levels Independently Affect Response Characteristics

**Figure 4:**
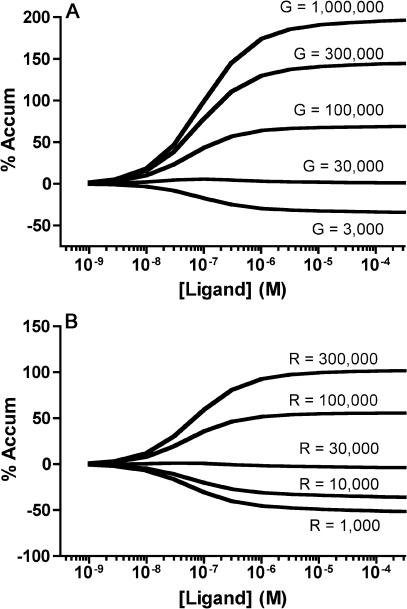
The Effect of Changing Receptor and G Protein Expression on the Activation of G Protein Dose response curves measuring the percent accumulation of Gα_GTP_ (%Accum) were calculated according to Equation 2 as described in Methods. (A) Total G protein (G_total_) is varied from 1,000,000/cell to 3,000 per cell, and R_total_ = 5,000/cell. (B) Total receptor (R_total_) is varied from 30,000 to 1,000 per cell, and G_total_ = 10,000/cell. Parameter values: k_1_ = 1 s^−1^, k_3_ = 1 × 10^7^ M^−1^s^−1^, k_11_ =1 × 10^−4^ (number/cell)^−1^s^−1^, k_Gact_ = 5 s^−1^, k_GTP_ = 1 s^−1^, k_G_ = 1 × 10^−4^ (number/cell)^−1^s^−1^, K_a_ = 1 × 10^−8^ M^−1^, K_g_ = 1 × 10^−4^ (number/cell)^−1^, K_act_ = 0.01, α = 5, β = 5, δ = 0.5, γ = 0.1, η = 0.1.

The levels of receptor and G protein expression were both identified as important parameters in response generation (Table 2). To investigate the impact that variations in these parameters have on the character of response generation, simulations varying R_total_ and G_total_ over their physiologic range were performed. To simulate (but not replicate) experiments that measure the accumulation of a signal over time, such as radioligand assays, the integral of Gα_GTP_ over the first 10 s of ligand binding was calculated and normalized to basal values with no ligand present and is designated %Accum according to Equation 2. Qualitatively similar results are obtained using the instantaneous number of Gα_GTP_ at either 5 s or 10 s after ligand binding (unpublished data). For certain sets of parameters values, particularly when α, δ, and γ ≠ 1, the %Accum upon ligand addition may vary from positive to negative values. The explanation for this protean agonist behavior is as follows. In conditions of constitutive activity (L = 0, Kact ≠ 0), there exist two receptor–G protein complexes (RG and R*G) of which only R*G can activate G protein. Upon ligand addition, the number of receptor–G protein complexes increases to four (RG, R*G, LRG, and LR*G), two of which (R*G and LR*G) can activate G protein. At low values of R_total_ and G_total_, the addition of ligand to the system can actually reduce G protein activation because of the redistribution of receptors and G proteins among their various states. When this occurs, the ligand behaves as an inverse agonist; a decrease in the %Accum upon ligand addition is then seen (Figure 4A and 4B). As the total numbers of receptors and G proteins are increased, the total number of R*G and LR*G generated upon ligand addition greatly outnumber those of R*G prior to ligand addition, and the ligand behaves as a positive agonist [50]. Figure 4A shows the behavior of %Accum when R_total_ is held constant at 5,000/cell and G_total_ is varied. Note that as G protein expression is increased, the same ligand is predicted to change from an inverse to a neutral to a positive agonist. Similar trends are seen when G_total_ is held constant at 10,000/cell and R_total_ is varied (Figure 4B).

**Figure 5:**
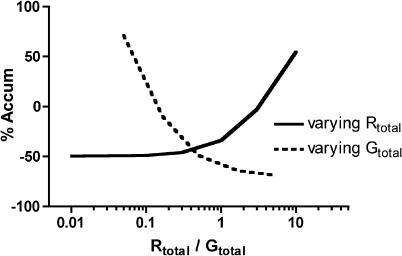
The Effect of the Ratio of Receptor to G Protein on G Protein Activation R_total_ was set at 5,000/cell and G_total_ varied (dashed line). As G protein expression increases, the response changes from negative to positive agonism. G_total_ was set at 100,000 per cell and R_total_ varied (solid line). As receptor expression increases, the response changes from negative to positive agonism.

Previous studies have proposed that the ratio of receptors to G proteins may play an important role in determining the efficacy of a ligand [40,51]. Furthermore, previous analysis of the equilibrium cTCM has shown that relatively large changes (>25-fold) in K_act_ and G_total_ may change the efficacy of a ligand, from positive to neutral or neutral to negative responses [50]. To investigate the role of the R/G ratio in our more physiological model, %Accum at a saturating ligand concentration (10 μM) from Figure 4A and 4B was plotted versus the ratio of R_total_/G_total_ in Figure 5. As seen in Figure 4, the level of response increased as either receptor or G protein levels were increased. However, because increasing G_total_ decreases the ratio R/G, the response generated in simulations of constant R_total_ and varying G_total_ (dashed line in Figure 5) increases as R_total_/G_total_ decreases. In contrast, as receptor expression is increased, the level of response and the ratio R_total_/G_total_ increase as indicated by a positive slope in the curve (solid line in Figure 5). Thus, there is not a clear relationship between the ratio R_total_/G_total_, and this measure cannot be used as a straightforward predictor of response efficacy. These results indicate that the individual levels of receptor and G protein expression are important in determining ligand efficacy.

### Our Results Offer New Explanations for Protean Agonism in the α_2A_- and β_2_-Adrenergic Receptor Systems

**Figure 6:**
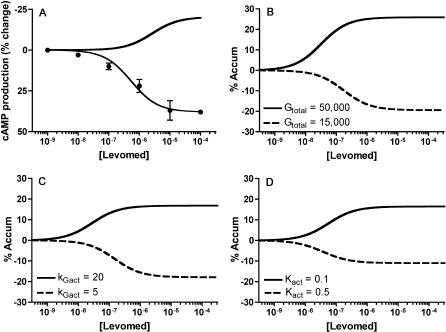
Protean Agonism in the α_2A_-Adrenergic System (A) Effect of levomed on cAMP production. Note that the *y*-axis in this plot is inverted from the usual to show positive agonists to have a positive slope and inverse agonists to have a negative slope. Levomed acts as an inverse agonist in HEL 92.1.7 cells (•, with curve fit). Data taken from Jansson et al. (1998), Figure 4. Levomed acts as a positive agonist in PC12 cells (line only). Data reconstructed from EC_50_ and max percent inhibition reported in Jansson et al. (1994), Table 1. (B–D) Simulations of protean agonism of levomed at the α_2A_-adrenergic receptor. Small changes in parameter values can cause the response to switch from positive to negative. (B) 3.3-Fold variation in G protein expression, β = 10. (C) A 4-fold variation in the G protein activation rate constant k_Gact,_ G_total_ = 100,000. (D) The equilibrium ratio of active to inactive receptors is varied 5-fold, G_total_ = 10,000, k_Gact_ = 5 s^−1^. Parameter values are equal to those listed in Figure 5 except when otherwise noted. R_total_ = 3,500 number/cell. Simulated dose response curves (B–D) measuring the percent accumulation of Gα_GTP_ (%Accum) were calculated according to Equation 2 as described in Methods.

α_2A_-Adrenergic receptors couple to Gα_i_ proteins, activating the G proteins, which in turn inhibit adenylyl cyclase activation. Jansson and colleagues have reported that the α_2A_-adrenergic ligand levomed is a positive agonist in PC10 cells, causing an inhibition of cAMP production as shown in Figure 6A [45]. However, the same group has reported that levomed also acts as an inverse agonist in HEL 92.1.7 cells, causing an increase in cAMP production (Figure 6A) [42]. These results suggest that a parameter (or parameters) different between the two cell types may play a critical role in determining the character of the response, and cause protean agonism.

Guided by the results from our uncertainty and sensitivity analysis (Table 2), we tested whether changes in a single cell–specific parameter, a parameter that might differ between the PC10 and HEL 92.1.7 cells, could produce protean agonism. Small (less than or equal to half an order of magnitude) changes in any of three parameters, G_total_, k_Gact_, or K_act_, were all able to produce protean agonism, as shown with representative simulations in Figure 6B–6D. All three changes represent physiologically reasonable explanations for the protean agonism. Differences in G protein expression (G_total_) between two cell lines and indeed within a cell line are common, although actual expression levels are rarely quantified [52–54]. Differences in the rate constant of G protein activation (k_Gact_) between cell lines then may be due to differences in G protein isoform expression profiles [55]. Further, differences in the equilibrium ratio of active to inactive receptors (K_act_) between cells expressing endogenous receptors and those with transfected receptors, in this case, between the transfected PC10 and endogenous HEL 92.1.7 cells studied by Jansson and colleagues, would also seem quite plausible.

Interestingly, although differences in α_2A_-adrenergic receptor expression between HEL 92.1.7 cells (2,900–4,100 receptors at the cell membrane; [56]) and PC12 cells (about 5-fold greater; [57]) have been reported, in our simulations these modest changes in R_total_ did not produce protean agonism. Similarly, although the GTPase activity of the Gα protein subunit (k_GTP_) can vary widely between different cell types depending on the expression of RGS proteins [3,25,58], variation of k_GTP_ did not produce protean agonism in our model. Other cellular parameters identified by uncertainty and sensitivity analysis (Table 2) also did not produce protean agonism, at least not for moderate (less than an order of magnitude) changes in their values and for physiologically reasonable values of the remaining parameters.

**Figure 7:**
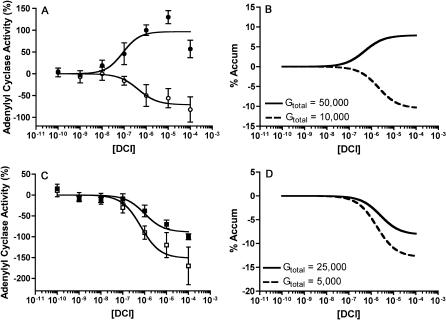
Protean Agonism in the β_2_-Adrenergic Receptor System (A) Dichloroisoproterenol (DCI) effect on adenylyl cyclase activity in Sf9 cells. DCI was found to be both a partial agonist (•) and an inverse agonist (○) in this study. Data replotted from Chidiac et al. (1996). (B) Simulations of DCI activation of Gα_GTP_. Protean agonism properties of DCI caused by 5-fold difference in G protein concentration. (C) After desensitizing treatment with isopreterenol, DCI was found to inhibit adenylyl cyclase activity in membranes where previously positive agonism was seen (•). This treatment further decreased activity of adenylyl cyclase in membranes where inverse agonism was observed (○). Data replotted from Chidiac et al. (1996). (D) Desensitization treatment by isopreterenol is simulated by decreasing G protein (G_total_) by 50%. Parameter values are equal to those listed in Figure 4 except when otherwise noted. R_total_ = 4,000/cell, α = 0.5, δ = 5. Simulated dose response curves (B,D) measuring the percent accumulation of Gα_GTP_ (%Accum) were calculated according to Equation 2 as described in Methods.

As a second example of protean agonism, Chidiac and colleagues have reported that the β_2_-adrenergic ligand DCI produces both stimulatory and inhibitory responses in Sf9 cells despite similar treatment of the cells (Figure 7A) [41]. The parameter with the most likely variation in this system is G protein expression (G_total_). While quantitative measurement of Gα_s_-like proteins in Sf9 cells has not to our knowledge been reported, several studies have shown that there may be wide variations in G protein expression in this system. Seifert et al. [52] and several references therein report that endogenous Gα_s_-like proteins could not be detected with immunoblot assays of Sf9 membranes, while Kleymann et al. [59] and Leopoldt et al. [54] have detected endogenous Gα_s_-like proteins in immunoblots of membranes. Additionally, infection of Sf9 cells with baculoviruses has been shown to downregulate the expression of endogenous G proteins [54]. Simulations at varying G_total_ showed that as little as a 5-fold change in G_total_ was able to produce protean agonism in our model, as shown in Figure 7B.

Chidiac and colleagues further report that after treatment with isoproterenol (a β_2_-adrenergic agonist) DCI acts as an inverse agonist only (Figure 7C) [41]. In other words, protean agonism by DCI is not seen after receptor desensitization. In another cell type (S49 lymphoma cells), Insel and colleagues have found that redistribution of Gα protein subunit between the membrane and cytosolic compartments occurs after prolonged treatment with isoproterenol [60]. The possibility that this loss of G proteins contributes to inverse agonism in Sf9 cells was tested by decreasing the number of available G proteins (G_total_). Ligand stimulation of these desensitized cells (parameters as in Figure 7B but with half the number of G proteins) produced only inverse agonism in our model (Figure 7D). Thus our analysis suggests the observation of positive or inverse agonism in these cells is sensitive to G protein number and that small changes in G protein number can account for observations of both protean agonism (before desensitization) and inverse agonism (after desensitization).

## Discussion

As is common in large signal transduction networks, there are significant uncertainties in model parameter values, and it is difficult to intuit model behavior. Using efficient parameter variation and sensitivity analysis methods, we identify parameters in a dynamic G protein activation model that play key roles in determining the level and indeed the character (positive/neutral/inverse agonism) of the response. This type of analysis allows for quick and efficient identification of important parameters. Drug design and development typically focus on altering ligand-specific parameters to produce desired cell response. Significantly, however, we find that not only ligand-specific parameters, but also cell-specific parameters, play critical roles in determining response behavior. To demonstrate how changes in cellular specific parameters can dramatically change cell response, we apply our findings towards studies of protean agonism in the α_2A_- and β_2_-adrenergic receptor systems.

Our analysis shows that several cell-specific parameters not previously identified contribute significantly to the character of ligand-induced responses. These include the total receptor concentration (R_total_), the GTP hydrolysis rate constant (k_GTP_), and the G protein activation rate constant (k_Gact_) (see Table 2 for a full list). Receptor number is known to vary widely in different cell types, and is regularly manipulated using transfection technologies. This is one of the few parameters in our model that is routinely quantified. It is well known that the GTPase activity of the Gα protein subunit may vary and is largely dependent on the presence of RGS proteins in the system [61,62]. Therefore, it is not surprising that the rate constant for GTP hydrolysis (k_GTP_) would be found to be important in response generation; indeed much attention has been given to RGS proteins as new therapeutic treatments [3,63,64]. Our findings are also consistent with an elegant study by Bornheimer and colleagues that analyzed G protein activation by active receptor and deactivation by GTPase activation proteins (GAPs) [12]. They found that local concentrations of receptors and GTPase activation proteins mediate various regimes of response behavior by kinetically controlling G protein activity. Following its identification by sensitivity analysis, we find that small changes in k_Gact_ (the rate constant for G protein activation) may result in protean agonism such as reported by Jansson and colleagues for the α_2A_-adrenergic system [42,45].

G protein concentration and K_act_, the ratio of active to inactive receptor states, are two cell-dependent parameters identified by our study (using a dynamic model of G protein activation) and two previous studies (using equilibrium models) as parameters that are key to determining the character of response and, when varied, could cause protean agonism (Table 2 and Figures 6B, 6D, and 7B) [20,40,50]. Overexpression or underexpression of receptor and G protein or expression of different isoforms of G proteins may give very different results than those found in endogenous systems. Additionally, G protein expression may vary largely in cells depending on a variety of factors, including cell type, state of cell development [65], prior treatments to the cells [54,60,66,67], and disease state [68–70]. Although K_act_ has not been rigorously quantified, receptor mutation and fluorescent imaging studies are lending valuable insight into how conformational changes in the receptor confer activity with and without ligand stimulus [71–75].

Our findings on the sensitivity of G protein activation to these parameters are corroborated by experimental studies implicating changes in G protein and receptor expression and receptor activity in cardiac and Alzheimer disease. For example, it has been shown that both β_1_- and β_2_-adrenergic receptors are expressed at decreased levels in studies of cardiac failure, while Gα_i_ subunits are increased [76]. As a second example, although muscarinic receptor density is not changed in brains of Alzheimer patients, the functionality of the receptors has been shown to be compromised (suggesting an inactive receptor state and changes in K_act_), and decreases in the function of Gα_q_ protein have also been reported [77]. Thus, both experimental reports and our modeling results suggest that small changes in these key parameters may disrupt normal signaling and lead to disease states.

To account for day-to-day and cell-to-cell variability, experimental results are almost always presented normalized to basal levels. However, clearly some information is lost when normalizing, both in experimental data and in modeling studies. For example, in experimental systems normalizing washes out variations in the “basal state” of the cells that may be an indicator of the current state of the cell or of future response characteristics. In the context of our model, un-normalized cell population or single cell data would allow for a more in-depth study of how parameters contribute to both basal and post-ligand treatment responses.

Our findings represent what may be only a small sampling of cellular parameters that influence ligand efficacy. Indeed, small variations in combinations of parameters could also lead to observations of protean agonism. For the sake of conciseness, these are not analyzed here. However, the methods introduced here provide a computationally efficient mechanism to begin to explore all possibilities. One limitation of this type of analysis, particularly PRCC analysis, is that it can only identify trends in certain directions within the given parameter space. Other analysis techniques for LHS sampling, such as subjective, differential sensitivity analysis, one-at-a-time design, and the adjoint method have been used, although these also have significant limitations [39]. Another limitation for using PRCC is that the system must be monotonic; however, this is easily checked by monitoring scatterplots, and there exist other global analysis methods by which to analyze the impact of parameter variation on model output. Recent work has compared multiple global analysis methods and found that LHS/PRCC, genetic algorithm approaches, Sobol's method, and Fournier amplitude sensitivity test (FAST) give very similar results [35].

Finally, both kinetic measurements and modeling will be important to our progress in understanding and manipulation of G protein activation. New technologies, such as those reported in yeast and HEK 293 cells using fluorescence resonance energy transfer (FRET) and bioluminescence resonance energy transfer (BRET) to monitor receptor–G protein and G protein subunit interactions [48,49] have the potential to quantify these interactions in real time. Modeling studies by our group and others [12,33,78–83] facilitate our understanding of the complexities involved in cell signaling and identify pathway interactions that are key to describing normal and pathological cell functions. The dynamic model that we present here, a general model of GPCR and G protein activation, can be easily expanded to include details of particular GPCR systems and phenomena such as receptor desensitization and internalization, processes that do not operate under steady state conditions. Further, as the components involved in signal transduction become better described and incorporated into signaling databases (e.g., SigPath [84], Alliance for Cell signaling [85], National Center for Genome Resources's PathDB [86]) and as more complex models of signaling pathways become possible, it will become increasingly important to systematically assess parameter uncertainty and quantify regimes of model behavior.

## Materials and Methods

### The cubic ternary complex activation model.

The cubic ternary complex activation model (cTCAM) (Figure 1) is a kinetic extension of the (equilibrium) cTCM integrating a feedback loop of G protein activation and recycling with dynamic LRG interactions [22]. Each reaction in the model is governed by mass action kinetics. Reaction rate constants describe the association and dissociation of ligand and receptor (top to bottom of the cube) and receptor and G protein (front to back of the cube) as seen in Figure 1. The interconversion of inactive and active receptor states is described by forward and backward rate constants as viewed from left to right of the cube.

The cTCAM has a total of 27 kinetic rate constants, 24 describing the binding and dissociation reactions between ligand, receptor, and G protein, and three describing the G protein activation cycle. Additionally, the total concentrations of ligand, receptor, and G protein are required, bringing the total number of model parameters to 30. As described in Text S1, the model can be reduced to 16 input parameters (plus ligand concentration). A description of all the parameters used in the cTCAM is presented in Table 1. Briefly, four of these are ligand-specific parameters that control the binding of ligand to the receptor and the ability of ligand to bias receptor activation and G protein association (K_a_, k_3_, α, and γ, respectively). The eleven cell-specific parameters in the model determine the strength of association of the inactive and active receptor states with G protein (K_g_, k_11_, η, and β), the ratio of active to inactive receptor (k_1_, K_act_), the kinetics of G protein activation, deactivation, and recombination (k_Gact_, k_GTP,_ and k_G_), and the numbers of G proteins and receptors (G_total_, R_total_). The remaining constant, δ, is both a ligand- and cell-specific parameter that governs the synergistic effects between ligand binding, receptor activation, and G protein association [11].

The equations describing the cTCAM are presented in Text S1. To calculate the initial conditions for each species in the model under varying parameter values, the total receptor and G protein concentrations (R_total_ and G_total_) were set as the initial values of R and G while the initial values of all other species were set to zero. The system was allowed to come to steady state (typically less than 100 s) at which point a bolus of ligand (L) was added to the system (denoted as time = 0), and the formation of each species was tracked over time.

### Model analysis.

The level of Gα_GTP_ is the key output of the model. However, experimental data are routinely normalized to basal values (i.e., the amount of activated G protein in the absence of ligand). Therefore, for the model G protein activation was quantified as the percent change in Gα_GTP_ from basal (L = 0) as given by

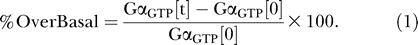
Quantifying response in this way is directly related to the pharmacological classifications of ligand efficacy—positive (increase in %OverBasal), neutral (no change in %OverBasal), and inverse agonism (decrease in %OverBasal)—and is analogous to that previously used to analyze response activation in the cTCM and cTCAM models [20,22,81].

Dose response curves were generated by calculating the integral of Gα_GTP_ (∫Gα_GTP_[t] dt) over the first 10 s of ligand stimulation at varying ligand concentrations. These dose-response curves are used to look at how responses may change upon varying receptor and G protein totals (Figure 4) and for comparison with experiments that measure the accumulation of radioligand, for example [^3^H]cAMP accumulation in studies of protean agonism in β_2_-adrenergic and α_2A_-adrenergic receptors (Figures 6 and 7). Values of the integral were normalized to basal values according to

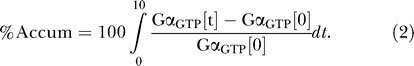
To distinguish this measurement from %OverBasal, this analysis is termed “%Accum.”

### Uncertainty and sensitivity analysis using Latin hypercube sampling and partial rank correlation coefficient.

To efficiently sample the ranges over which input parameters (x_i_, i = 1,2,..,X) may vary, LHS was implemented for the cTCAM using methods described previously [29,30,87,88]. Briefly, each input parameter is assigned a range according to values found in the literature (Table 1). Each parameter's value range was divided into N equal probable segments according to a specified probability distribution function for that parameter. For a typical simulation, N = 1,000. Distributions for most of these parameters are not known and therefore uniform distributions were used for each parameter. A random value was then chosen from each segment, so that each parameter became a vector of N values. The N values of the parameter vectors were then randomly paired to generate an N by X input matrix where X was the number of parameters to be varied (X = 16 for the cTCAM). The differential equations describing the model (Text S1) were then solved, generating a vector of N solutions.

PRCCs were calculated to quantify the relative importance of each parameter in generating a desired output (measures of Gα_GTP_ described above). Partial correlation measures the strength of the linear relationship between the output and an input variable (x_i_) after the effect of all other elements of **x** have been removed [38], while the rank transformation is used to linearize the nonlinear monotonic relationship between the input parameters and the output [29]. PRCC values vary between −1 (perfect negative correlation) and 1 (perfect positive correlation). PRCC values were calculated as described previously [29,30,87,88]. Briefly, solutions of Gα_GTP_ at desired time points were added to the LHS input matrix to generate an N by X + 1 matrix. The values for each of the X + 1 parameters were then ranked from 1 to N and the resulting matrix was used to calculate a partialized matrix in which the linearized effects of the other parameters are taken out of each parameter. Correlation coefficients were then calculated from the partialized matrix. Scatterplots were generated to assure that the monotonicity assumption applies [29]. The calculated PRCC values were differentiated based on *p*-values derived from a Student's *t* test and were then ranked according to their absolute value.

## Supporting Information

Figure S1 The Cubic Ternary Complex Activation Model with Rate Constants
(77 KB TIF)

Text S1 Click here for additional data file. Cubic Ternary Complex Activation Model
(93 KB DOC)

### Accession Numbers

The Swiss-Prot (http://ca.expasy.org) accession numbers for the proteins mentioned in the text are: α_2a_-adrenergic receptor (P08913), β_2_-adrenergic receptor (Q6GMT4), and Gα protein (Q6B6N3).

## Abbreviations

cTCM: cubic ternary complex model
DCI: dichloroisoproterenol
G: G protein
G protein: guanine nucleotide binding protein
Gα_GTP_: active G protein
GDP: guanosine diphosphate
GPCR: G protein–coupled receptor
GTP: guanosine trisphosphate
L: ligand
levomed: levomedetomidine
LHS: Latin hypercube sampling
LR: ligand–receptor
LR*: ligand-active receptor
LR*G: ligand-active receptor–G protein
LRG: ligand–receptor–G protein
PRCC: partial rank correlation coefficients
R: receptor
R*: active receptor
RGS: regulator of G protein signaling
TCM: ternary complex model
