# Supplementary material for: Both Ligand- and Cell-Specific Parameters Control Ligand Agonism in a Kinetic Model of G Protein–Coupled Receptor Signaling
Source: PLoS Comput Biol. 2007 Jan 12;3(1):e6. doi: 10.1371/journal.pcbi.0030006 (PMC1769407; doi:10.1371/journal.pcbi.0030006)
Supplement: Supplementary file 1 [file pcbi.0030006.sd001.doc]

Both Ligand- and Cell-Specific Parameters Control Ligand Agonism in

a Kinetic Model of G protein Coupled Receptor Signaling

by

Tamara L. Kinzer-Ursem and Jennifer J. Linderman

**Supporting Text - Cubic Ternary Complex Activation Model (cTCAM)**

The reactions and the accompanying rate constants necessary to describe the cTCAM are shown in Fig. S1. Model detail and assumptions are explained in this supporting text. Differential equations describing the cTCAM were written according to mass action kinetics and are also outlined below (Eqns. S.19 – S.30).

*G-protein activation/inactivation steps*

Under physiological conditions, activation of G protein by an active receptor (R*) initiates a multi-step process of GDP dissociation, GTP association, and dissociation of the G protein heterotrimer into Gα and Gβγ subunits. These elementary processes are represented in the cTCAM (Fig. 1 and A1) as a single step with active receptor-G protein complexes (R*G and LR*G) activating G protein with the rate constant k_Gact_ in accordance with findings that show the dissociation of GDP from the Gα protein subunit to be the rate limiting step [1,2]. It is assumed that rate constant of G protein activation (k_Gact_) is the same or similar for both constitutively active receptor/G-protein complexes (R*G) and ligand-associated active receptor/G protein complexes (LR*G). Gα_GTP_ is considered to be the response signal although it is known that Gβγ also couples to and activates signal transduction proteins [3,4]. The active Gα_GTP_ subunit is deactivated when the nucleotide is hydrolyzed to form Gα_GDP_ with a rate constant k_GTP_. This rate constant can vary over several 100 fold depending on the intrinsic GTPase activity of the Gα subunit or the presence of RGS proteins in the system [5,6]. Recombination of the Gα_GDP_ and Gβγ subunits to form free G protein occurs with rate constant k_G_.

*Assumptions regarding rate constants describing ligand, receptor, and G-protein interactions*

When the system is at equilibrium (i.e. k_Gact_=0) the principle of microscopic balance dictates that the forward and backward velocities of each elementary step must be equal [7,8]. This principle implies that the free energy change clockwise and counterclockwise around a path must be equal. For example, for the front face of the cube (Fig. S.1) the rate constants must be set so that k_1_k_5_k_8_k_4_=k_3_k_7_k_6_k_2_. Due to this thermodynamic constraint, only 19 of the 24 rate constants describing the ligand-receptor-G protein interactions are independent. Therefore, it is assumed that the ratio of the forward and backward rate constants in the model should reduce to the seven equilibrium constants of the cTCM ([9] and Fig. 1). These consist of three cellular dependent parameters (K_act_, K_g_, and β) that dictate receptor and G protein interaction in absence of ligand, three ligand dependent parameters (K_a_, α, and γ), and one parameter (δ) that is both ligand- and cell-specific. These relationships are

(S.1)	k_1_/k_2_ = K_act_

(S.2)	k_3_/k_4_ = K_a_

(S.3)	k_5_/k_6_ = αK_a_

(S.4)	k_7_/k_8_ = αK_act_

(S.5)	k_9_/ k_10_ = K_g_

(S.6)	k_11_/k_12_ = βK_g_

(S.7)	k_13_/k_14_ = βK_act_

(S.8)	k_15_/k_16_ = γK_g_

(S.9)	k_17_/k_18_ = βγδKg

(S.10)	k_19_/k_20_ = αβγKact

(S.11)	k_21_/k_22_ = αγδK_a_

(S.12)	k_23_/k_24_ = γK_a_

By specifying these equilibrium parameters (7 in total), then, if the forward rate constant is known, the reverse rate constant can be calculated, leaving 12 parameters to specify. The three forward rate constants k_1_, k_3_, and k_11_ will be specified. Assumptions for the remaining 9 rate constants can be grouped into and are presented below in three categories. The independent ligand- and cell- specific parameters that make up the cTCAM are summarized in Table 1.

*Rate constants describing the reversible association of ligands and receptors*

It is assumed that G protein binding does not affect the ligand association rate constant such that the rate constants for L+R→LR and L+RG→LRG are equal (k_3_=k_23_), and L+R*→LR*, L+R*G→LR*G are equal (k_5_=k_21_). In at least one particular system it has been shown that the association rate constants for ligand binding to R and RG are the same [10]. Ligand association with the active receptor state R* is assumed to be influenced by the ligand-specific parameter α such that k_5_ = αk_3_. Ligand-receptor dissociation rate constants are assumed not to be affected by receptor conformation such that the reactions LR→R and LR*→R* have equal rate constants (k_4_=k_6_). These assumptions are summarized in the following equations:

(S.13)	k_3_ = k_23_

(S.14)	k_5_ = k_21_ = αk_3_

(S.15)	k_4_ = k_6_

*Rate constants describing the reversible association of receptors and G proteins*

It is assumed that the inactive receptor conformation is unable to couple with full efficiency to G protein. The collision efficiency (η), an adjustable parameter ranging 0 to 1, is used to implement this assumption [11] such that k_9_=ηk_11_. Ligand biases receptor conformation based on the parameter α; however it is assumed here that ligand does not have an effect on the association of either the active or inactive receptor conformation with G protein such that the rate constants for the reactions R→RG and LR→LRG are equal (k_9_=k_15_) and R*→R*G and LR*→LR*G are equal (k_11_=k_17_). Thus:

(S.16)	k_9_ = k_15_ = ηk_11_

(S.17)	k_11_ = k_17_

*Rate constants for the interconversion of receptor conformations*

The receptor inactivation is assumed not to be affected by ligand or G protein binding such that the rate constants describing the R*→R, LR*→LR, R*G→RG, and LR*G→LRG are equal. Thus:

(S.18)	k_2_ = k_8_ = k_14_ = k_20_

*Model equations*

(S.19)	dR[t]/dt = -k_1_R[t] + (k_1_/K_act_)R*[t] - (ηk_11_)R[t]G[t] + ((ηk_11_)/K_g_)RG[t] – k_3_R[t]L + (k_3_/K_a_)*LR[t];

(S.20)	dLR[t]/dt = -(k_3_/K_a_)LR[t] + k_3_L*R[t] - αk_1_LR[t] + k_1_/K_act_LR*[t] – ηk_11_LR[t] G[t] + (ηk_11_/γK_g_)LRG[t]

(S.21)	dRG[t]/dt = ηk_11_R[t]G[t] - (ηk_11_/K_g_)RG[t] + (k_3_/γK_a_)LRG[t] - k_f_RG[t]L[t] - βk_1_RG[t] + (k_1_/K_act_)RG[t]

(S.22)	dLRG[t]/dt = k_3_RG[t]L - (k_3_/γK_a_)LRG[t] + ηk_11_LR[t]G[t] - (ηk_11_/γK_g_))LRG[t] + (k_1_/K_act_)LR*G[t] - k_1_αβδLRG[t]

(S.23)	dR*[t]/dt = k_1_R[t] - (k_1_/K_act_)R*[t] + (k_3_/K_a_)LR*[t] - (αk_3_)R*[t]L - βk_11_R*[t]
G[t] + (k_11_/K_g_)R*G[t] + k_Gact_R*G[t]

(S.24)	dLR*[t]/dt = αk_1_LR[t] - (k_1_/K_act_)LR*[t] - (k_3_/K_a_)LR*[t] + αk_3_R*[t]L - βk_11_LR*[t]G[t] + (k_11_/δγK_g_)LR*G[t] + k_Gact_LR*G[t]

(S.25)	dR*G[t]/dt = βk_11_G[t]R*[t] - (k_11_/K_g_)R*G[t] + βk_1_RG[t] - (k_1_/K_act_)R*G[t] -αk_3_R*G[t]L + (k_3_/δγK_a_)LR*G[t] - k_Gact_R*G[t]

(S.26)	dLR*G[t]/dt = αk_3_R*G[t]L - (k_3_/δγK_a_)LR*G[t] + βk_11_LR*[t]G[t] - (k_11_/δγK_g_)LR*G[t] + αβδk_1_LRG[t] - (k_1_/K_act_)LR*G[t] - k_Gact_LR*G[t]

(S.27)	dGα_GTP_[t]/dt = k_Gact_(R*G[t] + LR*G[t]) - k_GTP_ Gα_GTP_[t]

(S.28)	dGα_GDP_[t]/dt = k_GTP_ Gα_GTP_[t] - k_G_ Gα_GDP_[t]Gβγ[t]

(S.29)	dGβγ[t]/dt = k_Gact_(R*G[t] + LR*G[t]) - k_G_ Gα_GDP_[t]Gβγ[t]

(S.30)	dG[t]/dt = -ηk_11_R[t]G[t] + (ηk_11_/K_g_)RG[t] - ηk_11_LR[t]G[t] + (ηk_11_/γK_g_)LRG[t] - βk_11_LR*[t]G[t] + (k_11_/δγK_g_)LR*G[t] - βk_11_G[t]R*[t] + (k_11_/K_g_)R*G[t] + k_G_Gα_GDP_[t]Gβγ[t]

Equations were solved numerically using the NDSolve function in Mathematica® (Wolfram Research Inc., Champaign, IL). Total receptor and G protein (R_total_ and G_total_) were set as the initial values of R and G while all other species were set to zero. The system was allowed to come to steady state (typically less that 100 seconds) at which point ligand (L) was added (denoted as time = 0) and each species tracked over time.

**References**

1. Remmers AE, Engel C, Liu M, Neubig RR (1999) Interdomain interactions regulate GDP release from heterotrimeric G proteins. Biochemistry 38: 13795-13800.

2. Traynor JR, Clark MJ, Remmers AE (2002) Relationship between rate and extent of G protein activation: Comparison between full and partial opioid agonists. J Pharmacol Exp Ther 300: 157-161.

3. Robishaw JD, Berlot CH (2004) Translating G protein subunit diversity into functional specificity. Curr Opin Cell Biol 16: 206-209.

4. Cabrera-Vera TM, Vanhauwe J, Thomas TO, Medkova M, Preininger A, et al. (2003) Insights into G protein structure, function, and regulation. Endocr Rev 24: 765-781.

5. Lan KL, Zhong H, Nanamori M, Neubig RR (2000) Rapid kinetics of regulator of G-protein signaling (RGS)-mediated Galphai and Galphao deactivation. Galpha specificity of RGS4 and RGS7. J Biol Chem 275: 33497-33503.

6. Zhong H, Wade SM, Woolf PJ, Linderman JJ, Traynor JR, et al. (2002) A spatial focusing model for G protein signals: RGS protein-mediated kinetic scaffolding. J Biol Chem 278: 7278-7284.

7. Wyman J (1975) The turning wheel: A study in steady states. Proc Natl Acad Sci U S A 72: 3983-3987.

8. Lauffenburger DA, Linderman JJ (1993) Receptors: Models for binding, trafficking, and signaling. New York: Oxford University Press. 365 p.

9. Shea LD, Neubig RR, Linderman JJ (2000) Timing is everything - the role of kinetics in G protein activation. Life Sci 68: 647-658.

10. Posner RG, Fay SP, Domalewski MD, Sklar LA (1994) Continuous spectrofluorometric analysis of formyl peptide receptor ternary complex interactions. Mol Pharmacol 45: 65-73.

11. Shea LD, Omann GM, Linderman JJ (1997) Calculation of diffusion-limited kinetics for the reactions in collision coupling and receptor cross-linking. Biophys J 73: 2949-2959.

**Figure Legend**

**Figure S.1. The cubic ternary complex activation model (cTCAM).** The numbered (k_1_-k_24_) rate constants describe the forward and reverse reactions for ligand, receptor and G protein interactions, and the lettered rate constants (k_Gact_, k_GTP_, and k_G_) describe G protein activation and recycling [9].

**Figure S.1**
